# Supplementary material for: Transcription of HIV-1 at sites of intact latent provirus integration
Source: bioRxiv. 2024 Apr 29:2024.04.26.591331. Preprint. [Version 1] doi: 10.1101/2024.04.26.591331 (PMC11092494; doi:10.1101/2024.04.26.591331)
Supplement: Supplement 1 [file media-1.pdf]

Table S1. Clinical characteristics of study participants. Dx: diagnosis; ART: antiretroviral treatment; EFV: Efavirenz; TDF: tenofovir disoproxil fumarate; FTC: emtricitabine; DTG: dolutegravir.

ID	Age	Sex	Race	Year HIV-1 Dx	Year ART initiation	Years of uninterrupted ART	Viral Load (copies/mL)	$CD4^{+}$ T cell count	Reported Nadir	ART Regimen
603	43	Male	White/ Hispanic	12	10	10	<20	300	693	EFV/TDF /FTC
5104	35	Male	Black	7	7	7	<20	450	1006	DTG/TD F/FTC

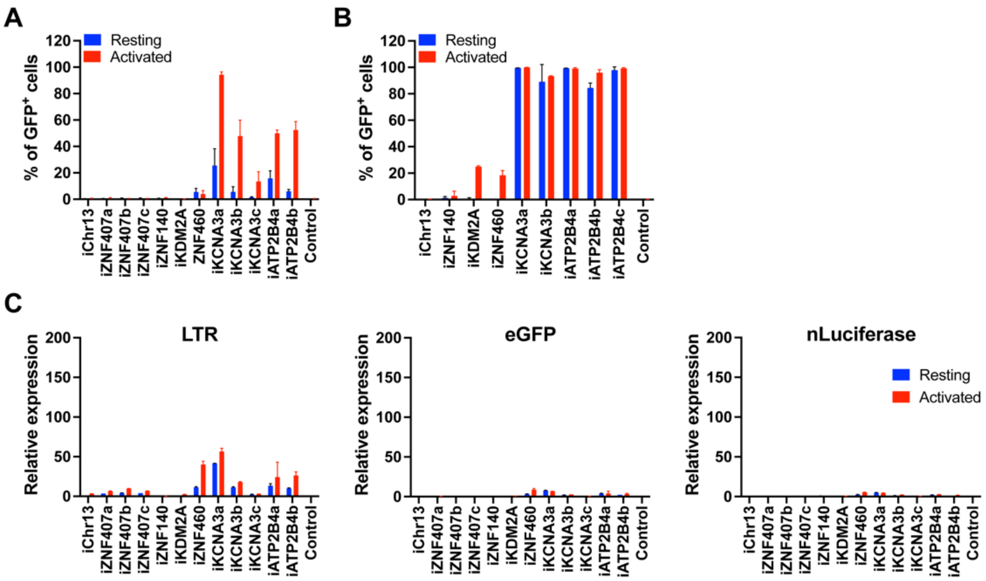

Figure S1. GFP expression in reporter cell lines. (A-B) Percentage of GFP+ cells as measured by flow cytometry for each integration-positive clone and control, under resting (blue) and PMA/ionomycin- and CD3/CD28-activated (red) conditions, respectively for Jurkat **(A)** and primary $CD4^+$ T **(B)** cells. Bars represent the mean of two independent experiments (biological replicates) ± standard deviation. **(C)** Graphs showing relative LTR (left panel), eGFP (middle panel) and nLuciferase (right panel) expression assessed by qPCR in Jurkat cell clones under resting (blue) and CD3/CD28-activated (red) conditions. Bars represent the mean relative expression from two independent assays (biological replicates) ± standard deviation.

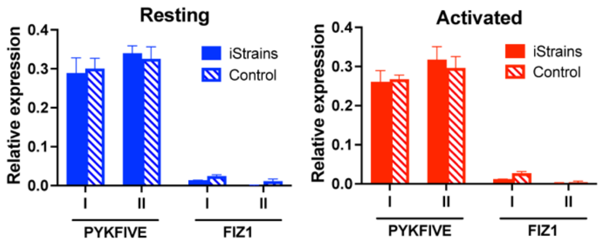

Figure S2. Expression host genes carrying reporter proviruses in Jurkat clones. Relative expression of host genes *PYKFYVE* and *Fiz1* in Jurkat clones (full bars) and control (stripped bars), under resting (blue) and activated (red) conditions. Bars represent the mean relative expression ± standard deviation for each gene for three technical replicates, for two independent assays (biological replicates**,** I and II).

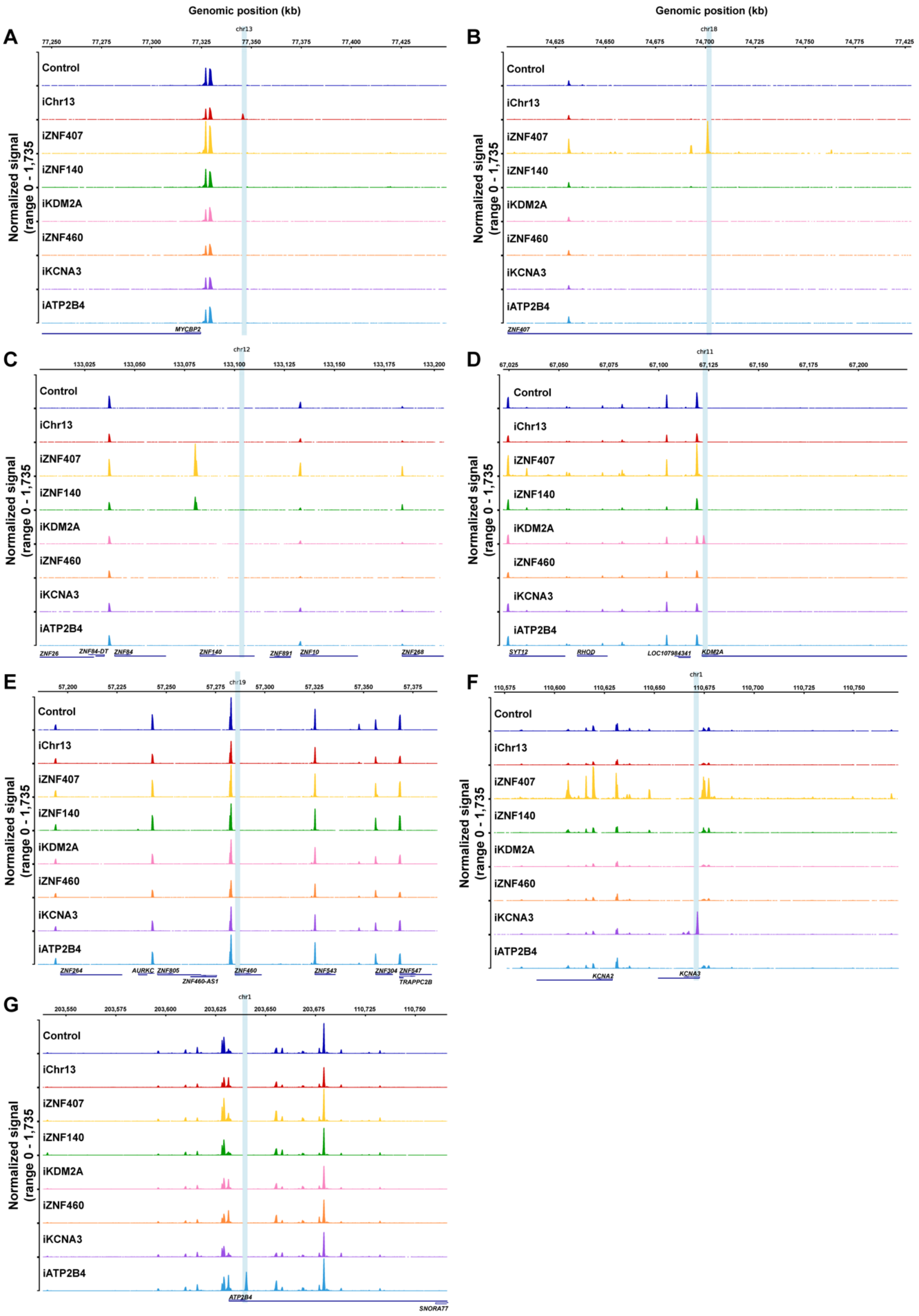
Genomic position (kb)
Genomic position (kb)
A
B
C
D
E
F
G
chr13
chr18
chr12
chr11
chr19
chr1
chr1
Normalized signal (range 0 - 1,735)
Control
iChr13
iZNF407
iZNF140
iKDM2A
iZNF460
iKCNA3
iATP2B4
MYCBP2
ZNF407
ZNF26
ZNF84-DT
ZNF84
ZNF140
ZNF891
ZNF10
ZNF268
SYT12
RHOD
LOC107984341
KDM2A
ZNF264
AURKC
ZNF805
ZNF460-AS1
ZNF460
ZNF543
ZNF304
ZNF547
TRAPPC2B
KCNA2
KCNA3
ATP2B4
SNORA77

Figure S3. Chromatin accessibility around reporter construct integration site in Jurkat cell clones. (A-G) Chromatin accessibility measured by ATAC-Seq in a 200,000 kb window of the genome around each of the integration sites for all Jurkat clones: chr13 (**A**), chr18 (*ZNF407*, **B**), chr12 (*ZNF140*, **C**), chr11 (*KDM2A*, **D**), chr19 (*ZNF460*, **E**), chr1 (*KCNA3*, **F**) and chr1 (*ATP2B4*, **G**). Graphs were generated by averaging the normalized reads from three technical replicates for each clone.

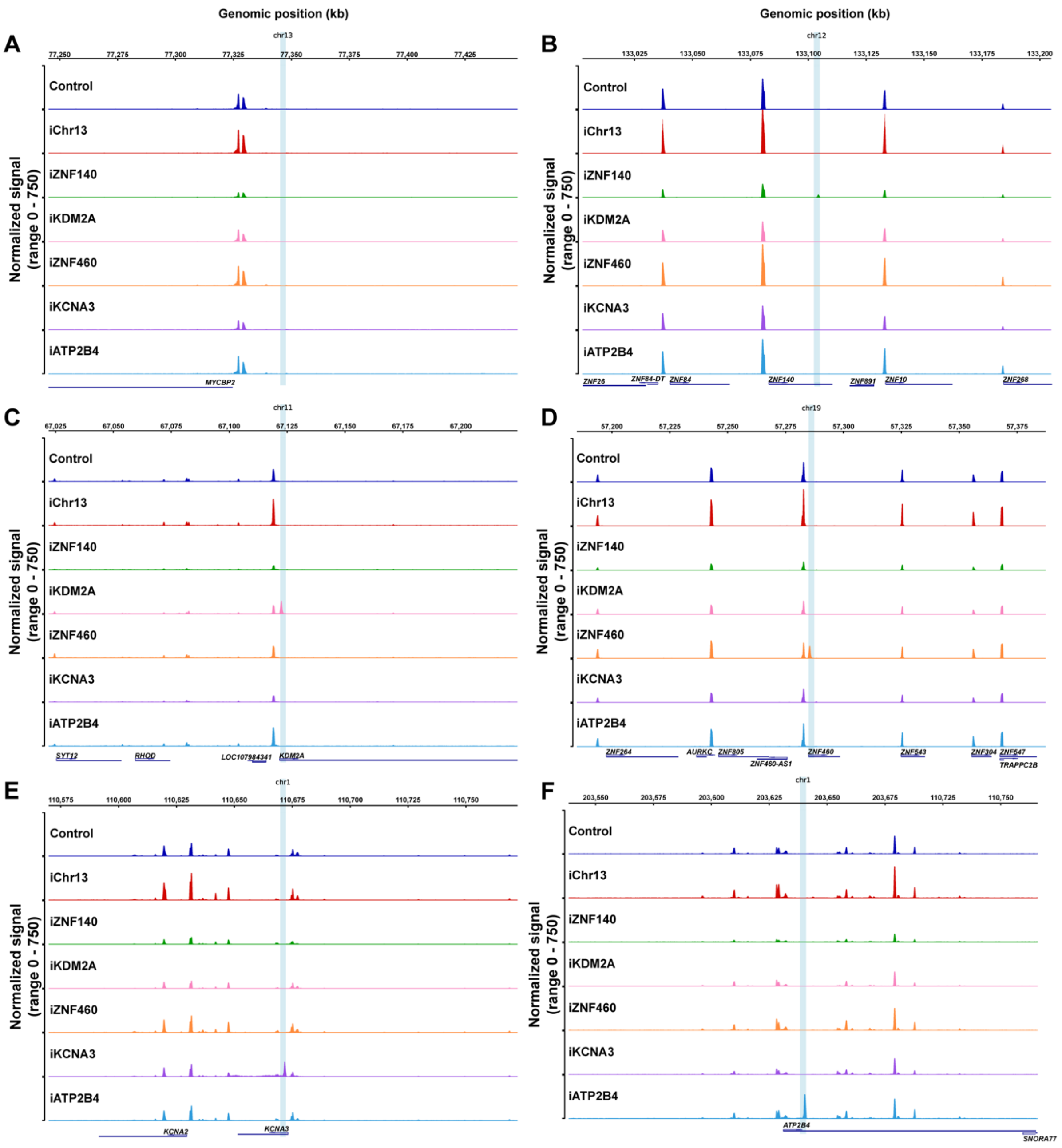

Figure S4. Chromatin accessibility around reporter construct integration site in primary CD4$^+$ T cell clones. (A-F) Chromatin accessibility measured by ATAC-Seq in a 200,000 kb window of the genome around each of the integration sites for all primary CD4$^+$ T cell clones: chr13 (**A**), chr12 (*ZNF140*, **B**), chr11 (*KDM2A*, **C**), chr19 (*ZNF460*, **D**), chr1 (*KCNA3*, **E**) and chr1 (*ATP2B4*, **F**). Graphs were generated by averaging the normalized reads from three technical replicates for each clone.

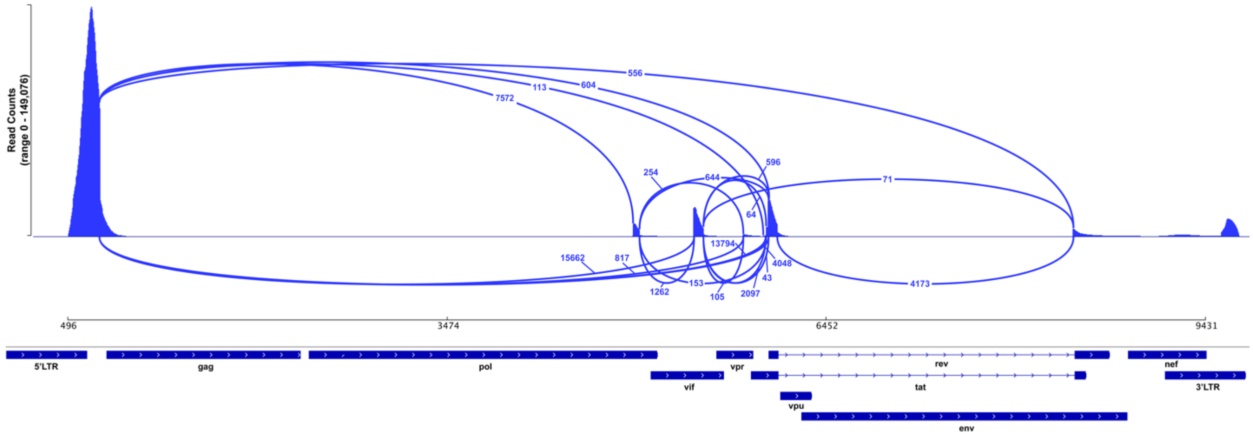

Figure S5. HIV-1 RNA splice variants. Sashimi plot showing scRNA-Seq reads of 10X single cell gene-expression data mapping to the HIV genome (bottom), in infected cells of participant 5104 under resting conditions. Splice junctions are represented as arcs connecting exons and the histogram represents the read coverage at each junction. The number of reads mapping to each junction is indicated by the numbers associated with each arc.
